# Association between moderate to severe psoriasis and periodontitis in a Scandinavian population

**DOI:** 10.1186/1472-6831-14-139

**Published:** 2014-11-26

**Authors:** Rasa Skudutyte-Rysstad, Ellen Margrethe Slevolden, Bjørn Frode Hansen, Leiv Sandvik, Hans Ragnar Preus

**Affiliations:** Institute of Clinical Dentistry, Faculty of Dentistry, University of Oslo, Oslo, Norway; The Dermatology Department, Oslo University Hospital-Rikshospitalet, Oslo, Norway

**Keywords:** Alveolar bone loss, Comorbidity, Periodontitis, Psoriasis

## Abstract

**Background:**

The aim of the present study was to compare the prevalence of periodontitis and alveolar bone loss among individuals with psoriasis and a group of randomly selected controls.

**Methods:**

Fifty individuals with psoriasis and 121 controls completed a structured questionnaire, and were examined clinically and radiographically. Oral examination included numbers of missing teeth, probing pocket depth (PPD), clinical attachment level (CAL), presence of dental plaque and bleeding on probing, as well as alveolar bone loss from radiographs. Questionnaires requested information on age, gender, education, dental care, smoking habits, general diseases and medicament use. For adjustment for baseline differences between psoriasis individuals and controls the propensity score based on gender, age and education was computed using multivariate logistic regression. A subsample analysis for propensity score matched psoriasis individuals (n = 50) and controls (n = 50) was performed.

**Results:**

When compared with controls, psoriasis individuals had significantly more missing teeth and more sites with plaque and bleeding on probing. The prevalence of moderate and severe periodontitis was significantly higher among psoriasis individuals (24%) compared to healthy controls (10%). Similarly, 36% of psoriasis cases had one or more sites with radiographic bone loss ≥3 mm, compared to 13% of controls. Logistic regression analysis showed that the association between moderate/severe periodontitis and psoriasis remained statistically significant when adjusted for propensity score, but was attenuated when smoking was entered into the model. The association between psoriasis and one or more sites with bone loss ≥3 mm remained statistically significant when adjusted for propensity score and smoking and regularity of dental visits. In the propensity score (age, gender and education) matched sample (n = 100) psoriasis remained significantly associated with moderate/severe periodontitis and radiographic bone loss.

**Conclusions:**

Within the limits of the present study, periodontitis and radiographic bone loss is more common among patients with moderate/severe psoriasis compared with the general population. This association remained significant after controlling for confounders.

## Background

Periodontitis is a chronic inflammatory infectious disease characterized by an immunologically moderated destruction of dental supporting tissues [1]. The disease affects more than 50% of adults in USA, and 5 – 10% are so severely affected that they will lose one or more teeth [2]. Psoriasis is a chronic, inflammatory, multi-system disease with predominantly skin and joint manifestations affecting approximately 2% of the general population. Plaque psoriasis is the most common form of the disease, affecting 80-90% of the patients [3]. The microscopic alterations of psoriatic plaques include an infiltration of immune cells in the dermis and epidermis, a dilatation and an increase in the number of blood vessels in the upper dermis, and a massively thickened epidermis with atypical keratinocyte differentiation. It has been suggested that the immune system plays an important role in the pathogenesis of psoriasis [4]. A long-standing focus on possible associations between periodontitis and general diseases [5] has recently led to several clinical and epidemiological studies, suggesting a link between these two chronic, inflammatory conditions.

Two recent epidemiological studies reported that periodontitis may be an independent risk factor for developing psoriasis. In a prospective study among nurses in the USA by NAKIB et al. [6], self-reported periodontal bone loss was shown to be associated with an increased psoriasis incidence. According to the study authors, data on a history of periodontal bone loss of nurses was collected by mailed questionnaires and used as a proxy for periodontal disease in the study. Similarly, increased risk for psoriasis was found among patients with chronic periodontitis diagnosis based on data from Health Insurance Database in Taiwan [7]. The few available clinical studies reported that individuals with psoriasis have a significantly higher number of missing teeth, more severe periodontitis and lower mean alveolar bone level than controls [8, 9]. Similarly, individuals with psoriatic arthritis had more clinical attachment loss compared to age and gender matched healthy controls [10], but, after adjustment for confounders [8, 10], none of the studies reported an association between clinical or radiographic periodontitis parameters and psoriasis.

One suggested biological explanation for the association between the two diseases is that psoriasis and periodontitis may be associated due to common underlying pathologic conditions [11–14]. Periodontal plaque has several polyclonal and mitotic factors that may trigger autoimmune antibodies which in turn may cause skin lesions or vice-versa. Characteristic for both psoriasis and periodontitis is an exaggerated immune response to the microbiota residing at the epithelial surface. For instance, dendritic cells (DC) are important for the initiation and regulation of both innate and adaptive immunity, and form a bridge between the two immune systems by trafficking from the epithelial barriers to the regional lymph nodes [15, 16]. In both psoriasis and periodontitis, DC may play an important role in driving the exaggerated immune response [4, 16, 17].

On the other hand, other important factors may play significant roles and confound the association between periodontitis and psoriasis [18]. Several studies have reported that psoriasis is associated with both physical and psychological comorbidity, and may therefore have a significant impact on the educational and work activities of the patients [19, 20]. Moreover, psoriasis patients have impaired health-related quality of life, which may lead to unhealthy lifestyle behaviours such as smoking, alcohol consumption, decreased physical activity and obesity [21]. Several of these factors are associated with increased risk of periodontitis [22]. Thus it is essential that relevant background and behavioural variables, which may confound the association between periodontitis and psoriasis, are taken into account, however, to our knowledge, no previous studies controlled for socioeconomic status (education) of participants or dental attendance.

Despite the increased focus on the topic during recent years, there are very few clinical studies on association between periodontitis and psoriasis and existing evidence is inconsistent. It might be speculated, that in case the two diseases are related, the association would be stronger in individuals with more severe psoriasis. Thus, the aim of the present study was to compare prevalence of periodontitis and alveolar bone loss among individuals with moderate to severe psoriasis and a group of randomly selected controls, and to assess whether the association is sustained after adjustment for confounding factors.

## Methods

The present study was approved by the Regional Committee for Medical and Health Research Ethics, (REC- South-East (#2010/401) and conducted from October 2011 until December 2012 at the Faculty of Dentistry, University of Oslo and the Dermatology Department, Oslo University Hospital-Rikshospitalet, Norway.

### Study participants

Sample size was determined based on the assumption that the expected prevalence of periodontitis is 30% among individuals with psoriasis compared to 10% among otherwise healthy controls [23]. The calculations were performed using Fleiss method at OpenEpi open source software [24]. Based on a significance level of 0.05, power of 90% and 1:2 ratio of cases to controls, it was decided to include 120 healthy controls and 60 individuals with psoriasis in the study. A higher number of controls compared to psoriasis individuals were selected in order to increase the study power.

Individuals with psoriasis were consecutively recruited from the Department of Dermatology, Oslo University Hospital-Rikshospitalet, Norway. Sixty five individuals received invitation to participate during their regular visit to the dermatological department or from the inpatient unit. Their psoriasis diagnosis was verified by a resident dermatologist (EMS) using Psoriasis Area and Severity Index (PASI) [25]. This index is based on the degree of erythema, infiltration and scaling (respectively: 0 = none, 1 = mild, 2 = moderate, 3 = severe, 4 = very severe) and the extent of involvement of four body skin areas (head, trunk, arms and legs). According to European consensus definitions, plaque psoriasis is graded into mild and moderate to severe disease [26]. Psoriasis is classified as mild if the PASI is below 10, and moderate to severe if it is 10 or above. Patients with a PASI score ≥10 or ongoing systemic or biologic treatment were considered to have moderate to severe psoriasis and received an invitation to attend for clinical and radiographic periodontal examination at the Faculty of Dentistry, University of Oslo.

The inclusion criteria were individuals 18 – 65 years of age, who had suffered from moderate to severe psoriasis for more than five years (see above definition). Persons with known familiar hypercholesterolemia, concomitant inflammatory diseases such as infections, autoimmune disorders, malignancies and pregnancy were excluded. After subsequent invitation by telephone, 51 accepted the invitation and attended the study (79% response rate). During the clinical examination one of the patients was found to be edentulous and was excluded, leaving 50 individuals with moderate or severe psoriasis for further analysis.

**Figure 1:**
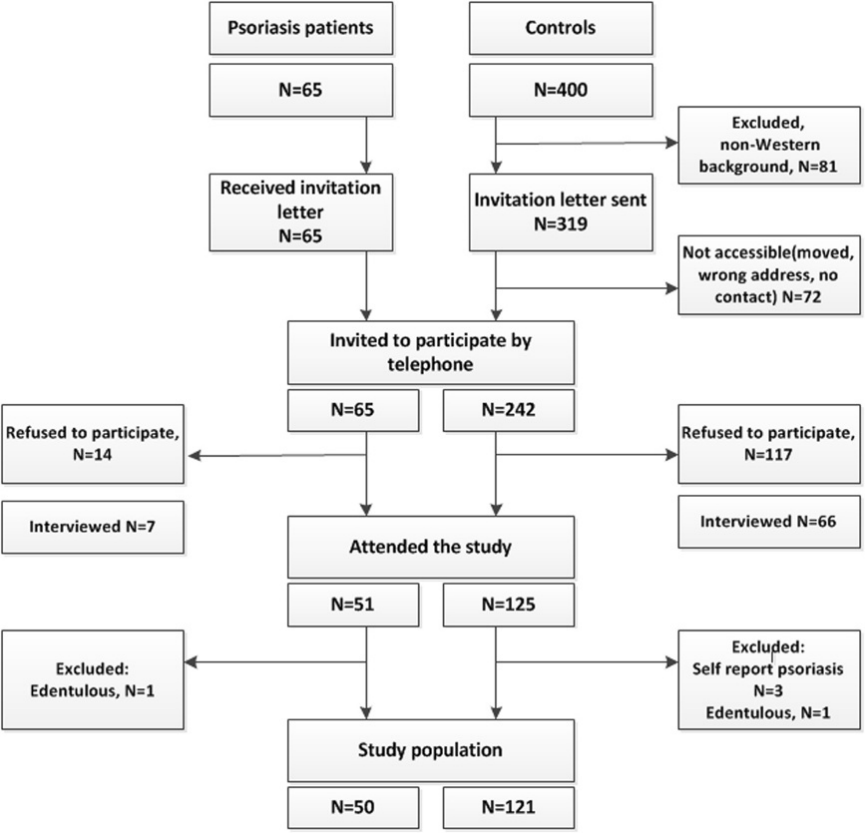
**Flow chart of the study population.**

The control sample was obtained from a list provided by the Norwegian National Population Register containing one thousand randomly selected 35-65-yr-old individuals, from the greater Oslo area. A smaller random sample of 400 individuals was selected in a two stage procedure (200 individuals at each stage) and invited to participate in the study. Only participants born in Norway or a Western country were selected. Of the 242 eligible individuals, 125 attended the study (response rate 52%) (Figure 1). After excluding three participants because of self reported psoriasis as well as one edentulous individual, the final study sample comprised 121 controls. Of those who refused to participate, 56% of controls and 50% of psoriasis individuals agreed to be telephone interviewed about their education, dental health related behavior, smoking habits and reason for not attending the study. Twenty per cent of the non-responders among controls reported that they had been diagnosed with gum disease. Comparisons of the participants and the non-attenders in relation to available background characteristics revealed that there were more current smokers among the non-responders in the control group. In contrast, a lower number of the non-attenders reported that they were current smokers (14% versus 32%) compared with the participants in the psoriasis group.

### Data collection

All participants received written information about the study and signed an informed consent. Data collection included self-administered questionnaires as well as clinical and radiographical examination at the Institute of Clinical Dentistry, Faculty of Dentistry. The structured questionnaire included background information (age, gender and education), information on dental care and dental attendance, smoking, self reported general diseases, and medicament use. All items in the questionnaire had closed ended questions, except for age, self reported diseases and medicament use, weight and height, which were open ended. Educational level was recorded as 1) lower secondary education or less, 2) upper secondary or 3) higher education. For regression analyses, the education variable was dichotomized into higher education versus upper secondary or lower education. Smoking status was recorded as never-, former- or current smokers. For regression analyses, the variable was dichotomized into current versus former and never smokers.

Clinical examination included numbers of missing teeth, assessment of probing pocket depth (PPD) and clinical attachment level (CAL) in mm, performed with an LM 52B probe (LM-Instruments Oy, Finland). PPD, CAL as well as the presence of dental plaque (yes/no) and bleeding on probing (BOP) (yes/no) were recorded at four surfaces (MB, B, DB, P/L) of all teeth present, except for the third molars. For statistical analyses, the recordings were computed into the proportion of sites with plaque or BOP. The clinical examination was performed by two trained examiners. The first examiner (RSR) recorded presence of plaque, BOP and PPD, whereas the second (BFH) recorded CAL.

Periodontitis was defined according to the case definitions for surveillance of periodontitis proposed by the Centers for Disease Control and Prevention (CDC) and the American Academy of Periodontology (AAP) [27]. Moderate periodontitis was defined as having ≥2 interproximal sites with AL ≥4 mm (not on same tooth), or ≥2 interproximal sites with PPD ≥5 mm (not on same tooth). Severe periodontitis was defined as having ≥2 interproximal sites with AL ≥6 mm (not on same tooth) and ≥1 interproximal site with PPD ≥5 mm. Individuals who had moderate or severe periodontitis according to the definition, were considered to have the disease.

Six intra-oral digital radiographs, two bitewings on each side and two periapical radiographs of the incisors of both jaws, were taken using Kwik-Bite bite-wing holders (Kerr Corporation, West Collins Orange, CA, USA) and Eggens (Eggen X-ray, Lillehammer, Norway) film holders. Radiographs were obtained by the Digora Digital imaging plate system film (Soredex, Finland ) and a Planmeca intra (Planmeca Oy, Finland) X-ray unit. The images were transferred and stored using Sectra PACS (Sectra AB, Linköping, Sweden) image server digital storage system. The radiographs were magnified using a computer, and radiographic bone loss was registered by one examiner (BFH) using the public domain digital imaging program Image J (Image J, National Institutes of Health, Bethesda, Maryland, USA). Bone loss was registered when the distance from the cemento-enamel junction (CEJ) to the alveolar crest (AC) exceeded 2 mm, in sites where CEJ and AC could be identified clearly. The measurements were made in mm in the interproximal areas of fully erupted canines, premolars, molars, upper central incisors and lower central and lateral incisors. Participants with radiographic bone loss ≥3 mm at one or more sites were considered as having the condition.

The radiographic examination was masked, i.e. the examiner was unaware of if participants had psoriasis or not, whereas the clinical examination could not be regarded as masked since some patients harbored psoriasis lesions visible to the examiner.

The intra-examiner reproducibility of radiographic bone loss was evaluated on the basis of repeat recordings radiographs from 20 randomly selected individuals within a time frame of 14 days , the *K*-value being recorded to 0.61 for recordings of bone loss in mm and 0.75 for dichotomized outcome (bone loss present/absent) and deemed as good [28].

### Statistical analyses

When comparing differences between the psoriasis and the control groups, the Chi-square test was used for categorical variables, and the independent samples t-test for continuous variables. In order to balance for differences in baseline characteristics between the psoriasis individuals and the controls, a propensity score was calculated for each person using logistic regression analysis, regressing psoriasis status (0 = no psoriasis; 1 = psoriasis) on the background characteristics (age, gender and education) [29]. The propensity score was considered as individuals’ probability of having psoriasis, conditional on the background covariates.

A subgroup of control individuals was then selected based on the propensity score values and 1:1 matched to individuals in the psoriasis group. This would allow to compare individuals who based on observables have more similar background characteristics (similar propensity score), except for their psoriasis status.

The association between psoriasis and periodontitis was investigated by using logistic regression analysis for the initial eligible study sample and for the propensity score matched sample. Variables significantly associated with the outcome in bivariate analyses at p-value <0.05, were entered into the regression analyses as independent variables. Multicollinearity diagnostics for independent variables was calculated using the Variance Inflation Factor (VIF) [30]. The VIF was <5 for all associations, indicating absence of multicollinearity which may invalidate the regression analysis. Results were reported as odds ratios (OR) with 95% confidence intervals (CI). The level of significance was set at 5%. Data were analyzed using the SPSS statistical program package (IBM SPSS 20.0, SPSS Inc., Chicago, IL, USA).

## Results

In the psoriasis group, mean disease duration was 22.3 years (range 4 to 52 years), and mean age was 45 years (range 22 to 64 years). The controls had a mean age of 49 years (range 35 to 66 years). The mean propensity score values were 0.51 (SD = 0.26) in the psoriasis group compared to 0.79 (SD = 0.17) in the control group (p < 0.001). After the selection of 50 controls most closely matching psoriasis individuals based on the propensity score, the mean propensity in the control group score was 0.63 (SD = 0.16), (p = 0.006).

Table 1 shows statistically significant differences between the psoriasis and the control group in relation to gender, education, oral hygiene, dental attendance, smoking habits and use of immunosuppressants. The majority of the participants with psoriasis were under medication at the time of investigation, and 68% reported use of immunosuppressive drugs, compared with 2% in the control group. As shown in Table 1, baseline characteristics of the psoriasis and control groups after propensity score matching were not significantly different, except for smoking and medicament use.

**Table 1:** **Background and behavioral characteristics of individuals with psoriasis and controls**

	All participants		Propensity score matched participants	
	Psoriasis individuals N = 50	Controls N = 121	Psoriasis individuals N = 50	Controls N = 50
**Age (yr)**				
Mean (SD)	44.4 (10.2)	48.6 (9.4)*	44.4 (10.2)	44.1 (8.1)
**Female gender**	24%	50%*	24%	32%
**Education**				
Lower secondary or less	16%	3%	16%	4%
Upper secondary	58%	28%	58%	54%
Higher education	26%	69%*	26%	42%
**Tooth brushing twice a day or more**	58%	85%*	58%	78%
**Daily interdental cleaning**	30%	41%	30%	32%
**Regular dental visits**	58%	89%*	58%	78%
**Last dental visit ≤1 yr ago**	62%	82%*	62%	72%
**Smoking**				
Never smoked	22%	47%	22%	52%
Former smoker	46%	40%	46%	30%
Current smoker	32%	13%*	32%	18%*
**BMI ≥ 25**	62%	51%	62%	60%
**Immunosuppressive medicaments**	68%	2%*	68%	2%*

*differences statistically significant at p < 0.05 level.

Three of the individuals with psoriasis had diabetes, whereof two had radiographic bone loss ≥3 mm. One individual in the control group had diabetes and radiographic bone loss ≥3 mm.

Participants with psoriasis had significantly higher mean numbers of missing teeth and higher mean proportions of sites with plaque and bleeding on probing compared with the controls (Table 2). The prevalence of periodontitis and the prevalence of radiographic bone loss was also significantly higher among individuals with psoriasis compared with the controls. Similarly, differences in clinical and radiologic findings were observed in the propensity score matched subsample.

**Table 2:** **Clinical and radiological findings in individuals with psoriasis and controls**

	All participants		Propensity score matched participants	
	Psoriasis individuals N = 50	Controls N = 121	Psoriasis individuals N = 50	Controls N = 50
**% of sites with PPD ≥4 mm**				
Mean (SD)	3.7 (4.1)	1.9 (3.8)*	3.7 (4.1)	2.2 (4.3)
**% of sites with PPD ≥5 mm**				
Mean (SD)	1.5 (3.3)	0.6 (1.9)*	1.5 (3.3)	0.6 (2.1)
**% of sites with CAL ≥ 2 mm**				
Mean (SD)	6.6 (9.5)	6.6 (8.8)	6.6 (9.5)	5.9 (6.7)
**Mild periodontitis, %**	18%	8%	18%	8%
**Moderate or severe periodontitis, %**	24%	10%*	24%	8%*
**% of sites with bone loss ≥3 mm**	4.6 (11.0)	0.9 (3.2)*	4.6 (11.0)	0.7 (2.3)*
Mean (SD)				
**One or more sites with bone loss ≥3 mm**	36%	13%*	36%	10%*
**Number of missing teeth**				
Mean (SD)	2.4 (3.9)	1.4 (2.5)*	2.4 (3.9)	1.1 (1.7)*
**% of sites with plaque**				
Mean (SD)	41 (25)	29 (20)*	41 (25)	33 (19)
**% of sites with bleeding on probing**				
Mean (SD)	37 (18)	24 (13)*	37(18)	26 (15)*

*differences statistically significant at p < 0.05 level.

Logistic regression analysis with adjustments for the propensity score for the whole sample was performed in order to assess whether the association between periodontitis and psoriasis remained significant when controlling for confounding. Two outcome variables were investigated: moderate/severe periodontitis and one or more sites with radiographic bone loss ≥3 mm. As shown in Table 3, the association between moderate/severe periodontitis and psoriasis remained statistically significant when adjusted for propensity score, but was attenuated when smoking was entered into the model. Adjustment for regularity of dental visits did not change the association. The association between psoriasis and one or more sites with radiographic bone loss ≥3 mm remained statistically significant when adjusted for propensity score and smoking and regularity of dental visits (Table 4). In the propensity score matched sample psoriasis remained significantly associated with moderate/severe periodontitis and radiographic bone loss (Tables 5 and 6).

**Table 3:** **Odds ratios (95% confidence interval) for the association of psoriasis and periodontitis, whole sample (N = 171)**

Model	OR (95% CI)	p	B	SE	Wald	df
**Unadjusted**	2.8 (1.2; 6.9)	0.019	1.054	0.450	5.493	1
**Model 1** ^**a**^	3.0 (1.0; 8.5)	0.043	1.089	0.538	4.0953.	1
**Model 2** ^**b**^	2.6 (0.9; 7.7)	0.079	0.963	0.548	090	1
**Model 3** ^**c**^	2.7 (0.9; 8.0)	0.069	1.002	0.552	3.296	1

^a^Model 1: logistic regression analysis using periodontitis as dependent variable and psoriasis status as independent variable, adjusted for propensity score.

^b^Model 2: as Model 1, except that smoking status was added as independent variable.

^c^Model 3: as Model 2, except that regularity of dental visits was added as independent variable.

Stepwise logistic regression analysis.

**Table 4:** **Odds ratios (95% confidence interval) for the association of psoriasis and radiographic bone loss, whole sample (N = 171)**

Model	OR (95% CI)	p	B	SE	Wald	df
**Unadjusted**	3.7 (1.7; 8.1)	0.001	1.306	0.399	10.739	1
**Model 1** ^**a**^	4.5 (1.7;11.5)	0.002	1.495	0.483	9.594	1
**Model 2** ^**b**^	4.0 (1.6; 10.5)	0.004	1.394	0.489	8.136	1
**Model 3** ^**c**^	4.0 (1.5; 10.4)	0.005	1.378	0.492	7.847	1

^a^Model 1: logistic regression analysis using radiographic bone loss as dependent variable and psoriasis status as independent variable, adjusted for propensity score.

^b^Model 2: as Model 1, except that smoking status was added as independent variable.

^c^Model 3: as Model 2, except that regularity of dental visits was added as independent variable.

Stepwise logistic regression analysis.

**Table 5:** **Odds Ratios (95% confidence interval) for the association of psoriasis and periodontitis, propensity score matched sample (N = 100)**

Model	OR (95% CI)	p	B	SE	Wald	df
**Unadjusted**	3.6 (1.1; 12.2)	0.037	1.290	0.618	4.361	1
**Model 1** ^**a**^	3.1 (0.9; 10.8)	0.071	1.143	0.632	3.271	1
**Model 2** ^**b**^	3.7 (1.0; 13.1)	0.047	1.297	0.652	3.960	1

^a^Model 1: logistic regression analysis using bone loss as dependent variable and psoriasis status as independent variable, adjusted for smoking.

^b^Model 2: as Model 1, except that that regularity of dental visits was added as independent variable.

Stepwise logistic regression analysis.

**Table 6:** **Odds Ratios (95% confidence interval) for the association of psoriasis and radiographic bone loss, propensity score matched sample (N = 100)**

Model	OR (95% CI)	p	B	SE	Wald	df
**Unadjusted**	5.0 (1.7; 15.1)	0.004	1.622	0.556	8.512	1
**Model 1** ^**a**^	4.6 (1.5; 13.9)	0.007	1.522	0.565	7.266	1
**Model 2** ^**b**^	4.5 (1.5; 13.7)	0.009	1.497	0.573	6.838	1

^a^Model 1: logistic regression analysis using bone loss as dependent variable and psoriasis status as independent variable, adjusted for smoking.

^b^Model 2: as Model 1, except that that regularity of dental visits was added as independent variable.

Stepwise logistic regression analysis.

## Discussion

In the present study, the prevalence of radiographic bone loss was significantly higher among psoriasis individuals than the randomly selected controls. This association remained statistically significant when adjusted for propensity score (age, gender and education) and controlling for smoking, scores and regularity of dental visits. Similarly, the prevalence of moderate to severe periodontitis was significantly higher among psoriasis patients, but this association was significant only in the propensity score (age, gender and education) matched subsample.

These results support previously reported findings, where individuals with psoriasis had significantly fewer teeth and reduced bone level compared to controls [8, 9]. However, contrary to the previous reports [8], the association between radiographic bone loss and psoriasis in the present study remained significant after controlling for confounders. One of the possible explanations for differences in the findings could be more severe degree of psoriasis among participants investigated in the present study.

The results of this study showed a significant association between psoriasis and radiographic bone loss, as well as the clinical periodontitis parameters. It may be suggested that while radiographic bone loss indicates a history of periodontal disease and reflects cumulative destruction over time, clinical periodontitis measurements are affected by current tissue inflammation levels, which on turn could be affected by smoking [31]. The fact that the association between moderate/severe periodontitis and psoriasis was attenuated when smoking was entered into the regression model for the whole sample supports this assumption.

Periodontitis is a cumulative condition and therefore radiographic bone loss, capturing past history of disease, was used in addition to the clinical registration of periodontal status. Clinical examiners were not calibrated which is a limitation of the present study. Contrary to the clinical registration, bone loss measurements were masked and therefore not exposed to the possibility that psoriasis history of participants could affect our measurements. According to the literature, the distance from the CEJ to the alveolar crest at sites without clinical loss of attachment ranges from 0.3 to 2.4 mm (mean 1.1, SD = 0.4), as measured on bite-wing radiographs [32]. In order to avoid misclassification of borderline values, bone loss ≥3 mm in interproximal sites was chosen as a key outcome. The risk of overestimation of bone loss on a specific site should therefore be eliminated. We chose to define bone loss on one site as bone loss caused by periodontitis, although in some rare instances there may be other local factors causing bone loss. On the other hand, because of unreadable sites, due to overlapping of teeth, margins of fillings and crowns etc., there is a risk of underestimating proximal periodontal bone loss on radiographs.

The cross-sectional design of the study precludes any causal interpretations of the observed associations. Since all the participants were of Norwegian or Western origin, generalization of our findings is limited to these populations. The response rate in our study was lower than anticipated, 79% in psoriasis group and 52% in control group. The comparisons of participants and non-responders of the control group indicate that the two groups were similar in socioeconomic, background and dental habits, but there were more smokers among the non-responders in the control group. According to Statistics Norway, 14% of 16-74-yr-olds in Oslo area in 2008-2012 are daily smokers, showing that the proportion of smokers in the control group was similar to the general population. In contrast, fewer non-attenders in the psoriasis group reported that they were current smokers. Since smoking affects periodontal health negatively [33], it can be speculated that the true difference in periodontal conditions between the psoriasis and control groups could be overestimated to some extent.

There were significant differences between psoriasis cases and controls in relation to background and behavioral variables. The propensity score adjustment was used in order to balance the two groups in relation to these differences. In addition to the adjustment in multivariate analyses, a propensity score matched analysis in a subsample of 100 participants was performed. By selecting 50 controls and matching them to 50 psoriasis individuals by their propensity score, the two groups were not statistically significant different in their background characteristics, except for smoking. Multivariate analyses for the whole sample and propensity score matched subsample yielded similar results.

Although in multivariate analyses we adjusted for several important confounders, the possibility of residual confounding cannot be ruled out. Therefore, larger population based studies using broader sets of possible confounders are warranted. Case-control studies of this type are also more prone to confounding if the cases and controls are selected from different populations. As patients at a hospital clinic, the cases were likely to have more severe psoriasis compared to general population of psoriasis patients, whereas the controls were selected from the general population and less likely to have health problems. To overcome this limitation, future studies should select cases and controls from the same population, such as patients with and without psoriasis at dermatology clinics.

The present study assessed periodontal conditions in individuals with moderate and severe psoriasis with long disease exposure time (mean 22 years), but, it remains uncertain whether our findings can be generalized to individuals with milder forms of psoriasis. Sixty-eight per cent of the psoriasis patients were using immunosuppressive medication. Several studies have shown that immunosuppressive drug therapy may reduce the inflammatory responses of the periodontal tissue to bacterial plaque [34]. However, in the present study, the anti-inflammatory effect of medicament use on periodontal conditions could not be seen. Psoriasis patients using immunosuppressive medication had slightly lower number of bleeding sites and prevalence of radiographic bone loss, but none of the differences were statistically significant. One of the possible explanations could be that the periodontal destruction is a cumulative condition, while use of medication was assessed at one time point. There is a possibility that the exposure time to immunosuppressive medicaments was too short to have an effect on periodontal destruction parameters. Due to the fact, that there were too few psoriasis patients not using immunosuppressive drugs, our study was not able to distinguish whether the observed differences could be attributed to the psoriasis or was a consequence of medicament use. Despite of that, our findings suggest that health personnel in contact with psoriasis patients, should be aware of and focus on periodontitis prevention in this group.

## Conclusions

Within the limits of the present study, results from the present study support the notion that periodontitis and radiographic bone loss are more common among patients with moderate/severe psoriasis compared with the general population. Thus these findings may imply a need for increased awareness and focus on periodontitis prevention for individuals with psoriasis.
